# Synergistic Anticancer Effects of Polyphyllin I and Evodiamine on Freshly-Removed Human Gastric Tumors

**DOI:** 10.1371/journal.pone.0065164

**Published:** 2013-06-07

**Authors:** Guofeng Yue, Jia Wei, Xiaoping Qian, Lixia Yu, Zhengyun Zou, Wenxian Guan, Hao Wang, Jie Shen, Baorui Liu

**Affiliations:** 1 The Comprehensive Cancer Centre, Drum Tower Clinical Medical College of Nanjing University of Chinese Medicine, Nanjing, Jiangsu, China; 2 The Comprehensive Cancer Centre, Drum Tower Hospital Affiliated to the Medical School of Nanjing University, Clinical Cancer Institute of Nanjing University, Nanjing, Jiangsu, China; 3 Department of Surgery, Drum Tower Hospital Affiliated to the Medical School of Nanjing University, Nanjing, Jiangsu, China; Bauer Research Foundation, United States of America

## Abstract

**Objective:**

The present study was designed to examine the anticancer effect of Traditional Chinese Medicine of polyphyllin I (PPI) and evodiamine (EVO) on freshly–removed gastric tumor tissues.

**Methods:**

Sixty freshly–removed gastric tumor tissues were collected. Their sensitivity to PPI, EVO, platinum (Pt), 5-FU, irinotecan (CPT-11) were determined by histoculture drug response assay (HDRA). Those samples were also formalin-fixed and paraffin-embedded, which were used to examine the mRNA expression levels of aprataxin(APTX), excision repair cross-complementing 1(ERCC1), thymidylate synthase(TS) and topoisomerase I(TOPO1) by quantitative RT-PCR. The association of the gene expression levels and *in vitro* sensitivity were analyzed.

**Results:**

PPI, EVO, Pt, 5-FU and CPT-11 had anticancer effects on the freshly-removed gastric tumor tissues with average inhibition rates of 20.64%±14.25% for PPI, 21.14%±13.43% for EVO, 50.57%±22.37% for Pt, 53.54%±22.03% for 5-FU, and 39.33%±24.79% for CPT-11, respectively. Combination of PPI and Pt, EVO and Pt, EVO and 5-FU had higher inhibition rates than any single drug of them (*P*<0.001, *P* = 0.028, *P* = 0.017, respectively). The mRNA expression levels of ERCC1 were correlated with Pt sensitivity (*rho* = −0.645, *P*<0.001); the mRNA expression levels of TS were correlated with 5-FU sensitivity (*rho* = −0.803, *P*<0.001). There were also weak but significant correlations between APTX mRNA expression levels and CPT-11 sensitivity (*rho* = −0.376, *P* = 0.017) or EVO sensitivity (*rho* = −0.322, *P* = 0.036). ERCC1 mRNA expression levels was markedly suppressed by the presentation of PPI (*P* = 0.001) and slightly suppressed by the presentation of EVO (*P* = 0.04); whereas, TS mRNA expression levels was markedly decreased by the presentation of EVO (*P* = 0.017) and slightly decreased by the presentation of PPI (*P* = 0.047).

**Conclusion:**

PPI and EVO both could inhibit the activity of freshly-removed gastric tumor, and they could enhance the anticancer effect of Pt and 5-FU by reducing the mRNA expression levels of ERCC1 and TS.

## Introduction

Gastric cancer remains one of the leading causes of cancer death worldwide [1], [2]. The standard first-line chemotherapy regimen for locally advanced or metastatic gastric cancer is platinum (cisplatin or oxaliplatin, Pt) and 5-FU combined with other drugs, including docetaxel and irinotecan (CPT-11) [3]; however, median survival remains meager – around one year. Personalized chemotherapy based on the mRNA expression of predictive biomarkers can maximize efficacy. Meanwhile, development of novel methods and potential anticancer drugs may improve the response rate and efficiency.

**Figure 1:**
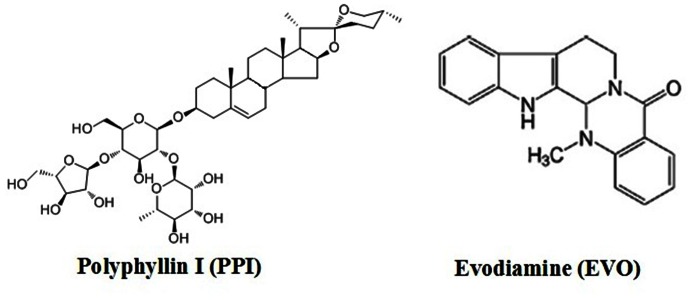
The chemical structure of PPI and EVO. PPI molecular weight: 855.02; molecular structure: C44H70O16. EVO molecular weight: 303.36; molecular structure: C19H17N3O.

Polyphyllin I (PPI), a small molecular monomer extracted from Rhizoma of Paris polyphyllin, is a steroidal saponin (Figure 1) [4]. In China, the rhizome of P. polyphylla, known as Chong-Lou, is reported to have effects on many tumor cells and xenografts, including the pancreas, urinary bladder, breast cancer, liver tumor and lung cancer, showing strong anticancer effects in previous studies[4]–[7].

Evodiamine (EVO) [8], a kind of alkaloid from Evodia rutaecarpa (Figure 1), has been reported to inhibit the invasion and metastasis of tumors and induces cell death in several types of cancer cell lines including human acute leukemia CCRF-CEM cells [9], human androgen independent prostate cancer PC-3 cells [10], human breast cancer MCF-7 cells [11], human melanoma A375-S2 cells [12], and murine fibrosarcoma L929 cells [13]. In addition, it has also been reported that EVO caused the mitotic arrest and a consequent apoptosis in CCRF-CEM cells through the enhancement of polymerised tubulin levels [14].

To date, studies demonstrating the anticancer activity of PPI and EVO have mainly been done with established cell lines. In the current study, we reported the cytotoxicity effect of PPI and EVO on freshly-removed gastric tumor tissues. To elucidate the mechanisms possibly involved, the expression levels of some associated genes were also determined.

## Materials and Methods

All research involving human participants have been approved by the Human Research Protective Committee of Drum Tower Hospital Affiliated to Medical School of Nanjing University and written informed consent was obtained from all patients.

### Patient Samples

**Figure 2:**
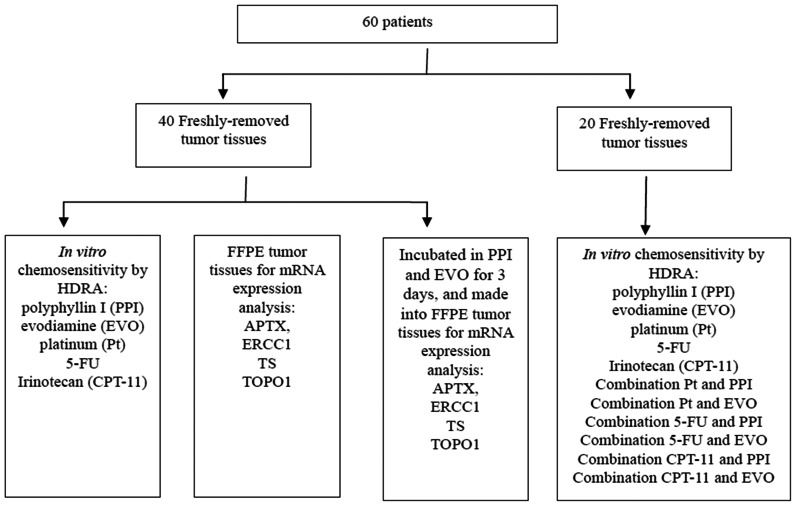
Flow chart showing patient disposition and experiments performed.

All specimens and relevant clinical data were obtained from the department of oncology and general surgery, Drum Tower Hospital Affiliated to Medical School of Nanjing University during the period from February 2012 to November 2012. The specimens included 60 freshly-removed gastric tumors. The study design is shown in Figure 2. Generally speaking, each tumor tissue was divided into two parts once it removed in the surgery: (1) one part was kept in 4°C Hanks’ balanced salt solution with 1% penicillin/streptomycin and detected chemosensitivity *in vitro* by histoculture drug response assay (HDRA); (2) the rest part was left in formalin and made into formalin-fixed paraffin-embedded (FFPE) tumor blocks for pathological observation and gene detection. Diagnosis of patients with gastric tumor was confirmed by histopathology. Clinical and histopathological data, including sex, histology, tumor site, stage, histological grade and lymph node metastasis were all collected. Clinical characteristics of the patients were summarized in Table 1.

**Table 1:** Patient characteristics.

Characteristic	Patients	Inhibition rate of PPI	Inhibition rate of EVO
	N = 40, N(%)	Mean ± Std	Mean ± Std
Age			
>61	20 (50%)	22.80% ±16.35%	23.29% ±13.90%
≤61	20 (50%)	18.25% ±11.46%	18.75% ±12.84%
Sex			
Male	32 (80%)	21.91% ±15.27%	21.99% ±13.64%
Female	8 (20%)	15.56% ±7.84%	17.70% ±12.83%
Histology			
Adenocarcinoma	28 (70%)	22.01% ±15.65%	21.38% ±13.52%
Mucinous	6 (15%)	18.28% ±10.87%	23.29% ±14.75%
Signet ring cell	6 (15%)	16.62% ±10.31%	17.83% ±13.56%
Tumor Site			
Distal stomach	11 (27.5%)	17.42% ±7.68%	16.78% ±11.06%
Proximal stomach	14 (35%)	16.13% ±16.45%	20.26% ±14.17%
Whole stomach	15 (37.5%)	27.21% ±14.05%	25.15% ±13.99%
Stage			
I,II	11 (27.5%)	21.27% ±13.51%	21.28% ±13.81%
III, IV	29 (72.5%)	20.40% ±14.75%	21.08% ±13.53%
Histological grade			
2	8 (20.0%)	18.31% ±17.22%	18.44% ±14.23%
3	27 (67.5%)	18.48% ±9.47%	22.23% ±9.90%
Mixed 2–3	5 (12.5%)	21.71% ±15.20%	21.31% ±24.54%
Lymph node metastasis			
No	3 (7.5%)	27.11% ±17.37%	22.97% ±24.06%
Yes	37 (92.5%)	20.12% ±14.12%	20.99% ±12.76%

### HDRA

**Figure 3:**
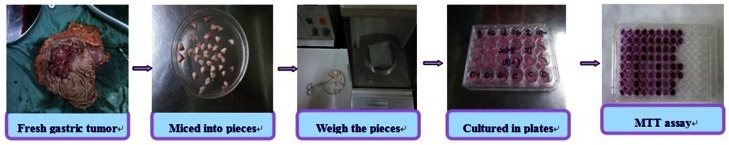
The procedure of histoculture drug response assay (HDRA).



HDRA procedures were performed as described previously [15]. The procedure of histoculture drug response assay was shown in Figure 3. Briefly, the fresh tumor tissues were washed and minced into small pieces to approximately 10 mg, which were then placed on prepared collagen surfaces in 24-well microplates. There were 8 parallel culture wells for each drug sensitivity testing and 8 parallel culture wells for control. After incubation for 7 days at 37°C (in a humidified atmosphere containing 95% air −5% CO_2_) in the presence of drugs dissolved with RPMI 1640 medium containing 20% fetal calf serum, 100 µl type I collagenase (0.1 mg/ml, Sigma) and MTT (5 mg/ml, Sigma) were added to each culture well and incubated for another 16 hours. Concentration of Pt, 5-FU, CPT-11, PPI and EVO was 20 µg/ml, 300 µg/ml, 20 µg/ml, 200 µg/ml and 200 µg/ml respectively, according to its peak plasma concentration (ppc) in patients [16]. Pt, 5-FU and CPT-11 were obtained from Jiangsu Hengrui Medicine Company (Jiangsu, China). PPI and EVO were supplied as powder with purity of >98% by the School of Pharmacy in China Pharmaceutical University. After extraction with dimethyl sulfoxide (DMSO, Sigma), absorbance of the solution in each well was read at 540 nm. Absorbance per gram of cultured tumor tissue was calculated from the mean absorbance of tissue from 8 parallel culture wells, and the tumor-tissue weight was determined before culture. The inhibition rate was calculated by using the following formula:

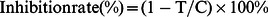
.

T is the mean absorbance of treated tumor /Weight.

C is the mean absorbance of control tumor / Weight.

### Synergy Analysis

Median effect analysis using the combination index (CI) method of Chou and Talalay [17] was employed to determine the nature of the interaction observed between PPI or EVO and chemotherapeutic agents. The CI is determined by the following equation: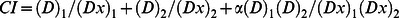, in which (*Dx*)_1_ and (*Dx*)_2_ are the concentrations for *D*_1_ (PPI or EVO) and *D*_2_ (chemotherapeutic agent) alone that gives *x*% inhibition rate, whereas (*D*)_1_ and (*D*)_2 _in the numerators are the concentrations of PPI or EVO and another drug that produce the identical level of effect in combination. 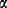 = 0 when the drugs are mutually exclusive, while 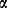 = 1 of they are mutually non-exclusive. CI >1 indicate antagonism, CI <1 indicate synergy, and CI = 1 indicate additivity.

### Total RNA Extraction from FFPE Tissue

Six 7-µm sections were prepared from FFPE tumor blocks that contained at least 80% tumor cells. After hematoxylin-eosin staining, the cancerous parts were microdissected and transferred into a microcentrifuge tube. RNA was isolated in accordance with a proprietary procedure (European patent number EP1945764-B1) [18]. Briefly, paraffin was removed by xylene, and microdissected cancerous parts were lysed in a proteinase K-containing buffer at 60°C for 16 h. RNA was purified by phenol and chloroform extractions followed by precipitation with isopropanol in the presence of sodium acetate at −20°C. The RNA pellet was washed in 70% ethanol and resuspended in 53 µl of RNase-free water followed by treatment with DNase I (Life Technologies).

### QPCR Assessment of Gene Expression

M-MLV Reverse Transcriptase Kit (Invitrogen) was applied to generate cDNA for Quantitative polymerase chain reaction (qPCR) to detect the β-actin (ACTB), aprataxin (APTX), excision repair cross-complementing 1 (ERCC1), thymidylate synthase (TS) and topoisomerase I (TOPO1). Each batch of reaction included a positive control from commercial human lung and liver RNA (Stratagene, La Jolla, CA, USA) as calibrators and negative controls without RNA and reverse transcriptase. Total RNA 1 µg was used for each RT reaction. Template cDNA was amplified with specific primers and probes for ACTB, APTX, ERCC1, TS and TOPO1 using Taqman Universal Master Mix (Applied Biosystems, Foster City, CA). The Assay IDs (Applied Biosystems, Foster City, CA) for the primers and probes were as follows: ACTB: Hs99999903_m1, APTX: Hs00214452_m1, ERCC1: Hs00157415_m1, TS: Hs00426586_m1, TOPO1: Hs00243257_m1. QPCR was performed to quantify gene expression using the ABI Prism 7900HT Sequence Detection System (Applied Biosystems). The PCR conditions were 50°C for 2 min, 95°C for 15 min, followed by 40 cycles at 95°C for 15 s and 60°C for 1 min. Relative gene expression quantifications were calculated according to the comparative Ct method using ACTB as an endogenous control, based on our previous experience comparing different housekeeping genes [18], [19], and commercial human lung and liver RNAs (Stratagene, La Jolla, CA, USA) as calibrators, which enables us to compare gene expression levels between different patients. Final results were determined by the formula mRNA expression level 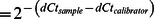 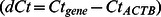 [18], [20] and were analyzed with the Stratagene analysis software.

### Statistical Analysis

Values were expressed as means ± standard deviation. Differences of inhibition rates between groups were evaluated using t-test. The Mann–Whitney U-test and the Kruskal-Wallis test were used to test the association between drug sensitivity or mRNA expression levels and clinical characteristics. The Spearman’s rank method was used to assess the correlation of the mRNA expression levels between different genes, and the correlation between mRNA levels and *in vitro* drug sensitivity. Paired Student’s t test was used to evaluate the differences of inhibition rates between before and after drugs incubation. A *P*<0.05 was considered statistically significant (two-sided). Statistical analysis was performed using the SPSS, version 16.0.

## Results

### Patient Characteristics and Sensitivity of Freshly-removed Tumor Tissues to Different Drugs

The method for sensitivity test of freshly-removed tumor tissues was successfully established and carried out (Figure 3). We first examined the cytotoxicity of each drug on freshly-removed tumor tissues of 40 testing samples, including PPI (200 µg/ml), EVO (200 µg/ml), Pt (20 µg/ml), 5-FU (300 µg/ml), CPT-11 (20 µg/ml).Characteristics of all patients are shown in Table 1. The mean age of those 40 patients was 61 (range: 34–81). The majority of patients were males (80%), and the majority histology of every sample was adenocarcinoma (70%). In 11 (27.5%) patients, the tumor was located in the distal stomach, in 14 (35%) in the proximal stomach, and in 15 (37.5%) in the whole stomach. Twenty-nine (72.5%) patients had stage III and IV disease. Lymph node metastasis was present in 37 (92.5%) patients.

**Figure 4:**
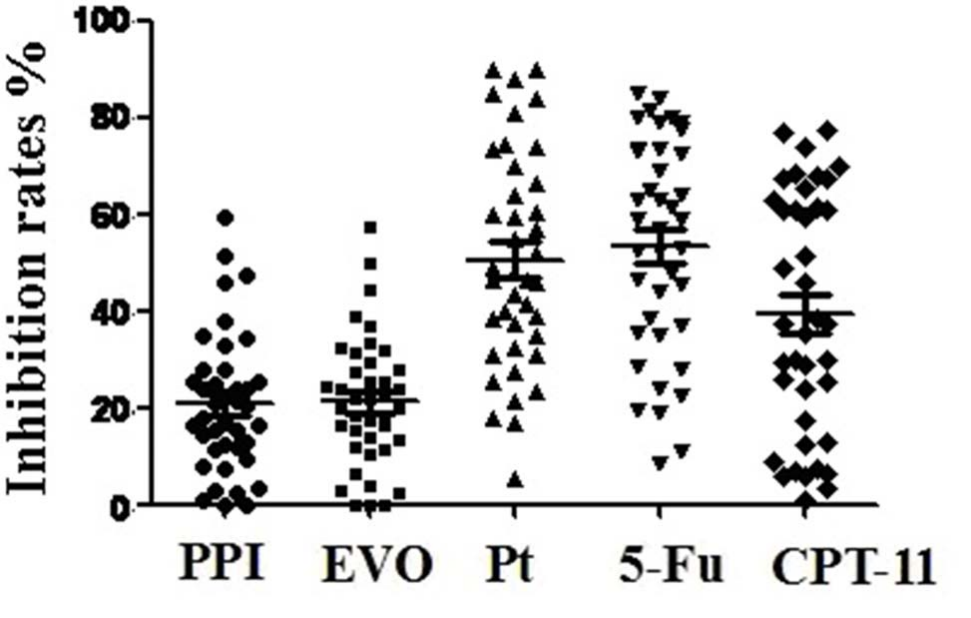
The inhibition rates of different agents on freshly-removed gastric cancer. **The lines in the middle stood for mean with SEM; n = 40.**

The Figure 4 shows the tumor inhibition rates for these tissues following exposure to the five agents. The average inhibition rate of the five drugs were 20.64%±14.25% for PPI, 21.14%±13.43% for EVO, 50.57%±22.37% for Pt, 53.54%±22.03% for 5-FU, and 39.33%±24.79% for CPT-11, respectively. It seems that although PPI and EVO exhibited certain anticancer effect, their efficiency were lower than chemotherapeutic agents (PPI vs. Pt:*P*<0.001;PPI vs. 5-FU:*P*<0.001;PPI vs. CPT-11: *P* = 0.013; EVO vs. Pt:*P*<0.001;EVO vs. 5-FU:*P*<0.001;EVO vs. CPT-11: *P = *0.037). There were no statistically significant differences in inhibition rates between PPI and EVO (*P*>0.05), and between different therapeutic agents (*P*>0.05). As shown in Table 1, there were neither no statistically significant differences in inhibition rates of PPI and EVO between age, gender, histology, tumor site, stage, or lymph node metastasis. Since the anticancer efficiency of PPI and EVO alone were not as significant as therapeutic agents, we further studied their effects on promotion of anticancer efficiency of therapeutic agents, and on reverse of chemoresistance on another 20 samples.

### Effects of PPI and EVO on Enhance the Anticancer Efficiency of Pt

**Figure 5:**
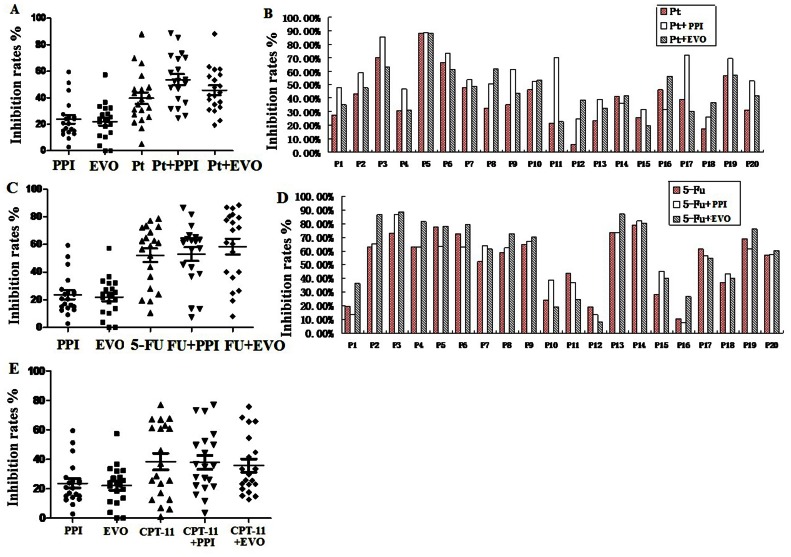
The inhibition rates of different agents or their combination on freshly-removed gastric cancer. A, combination Pt with PPI or EVO exhibits higher anticancer efficiency than any agents used alone (n = 20); B, the inhibition rates of every 20 samples after combination with Pt and PPI or EVO compared with Pt alone; C, combination 5-FU with PPI or EVO exhibits higher anticancer efficiency than any agents used alone (n = 20); D, the inhibition rates of every 20 samples after combination with 5-FU and PPI or EVO compared with 5-FU alone; E, combined with PPI or EVO, the inhibition rates of CPT-11 were not significant improved (n = 20).

As shown in Figure 5A, combination Pt (20 µg/ml) with PPI (200 µg/ml) or EVO (200 µg/ml) exhibited higher anticancer efficiency than any agents used alone. The average inhibition rate of the five regimen were 23.68%±14.32% for PPI alone, 22.08%±13.61% for EVO alone, 39.69%±19.57% for Pt alone, 53.56%±18.80% for Pt combined with PPI, 45.61%±16.29% for Pt combined with EVO, respectively. The inhibition rates of those 20 samples after combination with Pt and PPI or EVO compared with Pt alone were shown in Figure 5B. When combined with PPI, the inhibition rates were approximately 13.88% higher than Pt used alone (*P*<0.001, Paired Student’s t test) with maximal improvement reached 48.45%. When combined with EVO, the inhibition rates were approximately 5.92% higher than Pt used alone (*P* = 0.028, Paired Student’s t test) with maximal improvement reached 33.45%.

Some of the freshly-removed gastric tumor tissues in these samples were resistant to Pt with the inhibition rate below approximately 30%. Therefore, we examined the effect of PPI and EVO on reverse Pt resistance. Examples of Pt resistant samples were listed in Table 2 and Table S1, which showed that when Pt (20 µg/ml) combined with PPI (200 µg/ml) or EVO (200 µg/ml), resistant samples were sensitive to the treatment again with the inhibition rates more than 30% in general.

**Table 2:** The effects of PPI and EVO on reverse Pt resistance.

Patients	Inhibition rate of	Inhibition rate of	Inhibition rate of
	Pt(20 µg/ml)	Pt(20 µg/ml)+PPI(200 µg/ml)	Pt(20 µg/ml)+EVO(200 µg/ml)
P1	27.49%	47.61%	34.97%
P4	30.60%	46.70%	30.99%
P8	32.45%	50.86%	61.62%
P9	34.99%	61.12%	43.94%
P11	21.58%	70.03%	22.76%
P12	5.28%	24.53%	38.73%
P13	23.31%	39.17%	32.89%
P15	25.51%	31.41%	19.48%
P18	16.98%	26.34%	36.80%
P20	31.05%	52.89%	41.63%

### Effects of PPI and EVO on Enhance the Anticancer Efficiency of 5-FU

As shown in Figure 5C, combination 5-FU (300 µg/ml) with PPI (200 µg/ml) or EVO (200 µg/ml) exhibited higher anticancer efficiency than any agents used alone. The average inhibition rate of the five regimen were 23.68%±14.32% for PPI alone, 22.08±13.61% for EVO alone, 52.23±21.78% for 5-FU alone, 53.10%±21.91% for 5-FU combined with PPI, 58.49%±25.50% for 5-FU combined with EVO, respectively. The inhibition rates of those 20 samples after combination with 5-FU and PPI or EVO compared with 5-FU alone were shown in Figure 5D. When combined with PPI, the inhibition rates were approximately 0.87% higher than 5-FU used alone (*P*>0.05, Paired Student’s t test) with maximal improvement reached 17.36%. When combined with EVO, the inhibition rates were approximately 6.26% higher than 5-FU used alone (*P* = 0.017, Paired Student’s t test) with maximal improvement reached 23.63%.

The results of synergy analysis are summarized in Table 3, which shows, for each combination, the computer-calculated CI for 20, 50 and 80% inhibition rates, respectively. The CI values were below 1 in all combination at fraction affected **(**Fa) = 80%, indicating a synergistic anti-cancer effect. The CI values were also below 1 when combined PPI or EVO with Pt at Fa = 50%.

**Table 3:** Summary of CI values at 20, 50 and 80% fraction affected.

Regimen	CI at fraction affected (Fa)		
	20%	50%	80%
PPI+Pt	1.24	0.96	0.81
PPI+5-FU	1.49	1.15	0.9
EVO+Pt	1	0.92	0.96
EVO+5-FU	1.23	1.05	0.92

### Effects of PPI and EVO on Enhance the Anticancer Efficiency of CPT-11

As shown in Figure 5E, combination CPT-11 (20 µg/ml) with PPI (200 µg/ml) or EVO (200 µg/ml) exhibited higher anticancer efficiency than PPI or EVO used alone. The average inhibition rate of the five regimen were 23.68%±14.32% for PPI alone, 22.08±13.61% for EVO alone, 38.42%±25.19% for CPT-11 alone, 37.92±21.03% for CPT-11 combined with PPI, 35.71%±20.04% for CPT-11 combined with EVO, respectively. However, when combined with PPI or EVO, the inhibition rates of CPT-11 were not significant improved (*P*>0.05, Paired Student’s t test).

### mRNA Expression Levels of ERCC1, TS, TOPO1 and APTX

**Figure 6:**
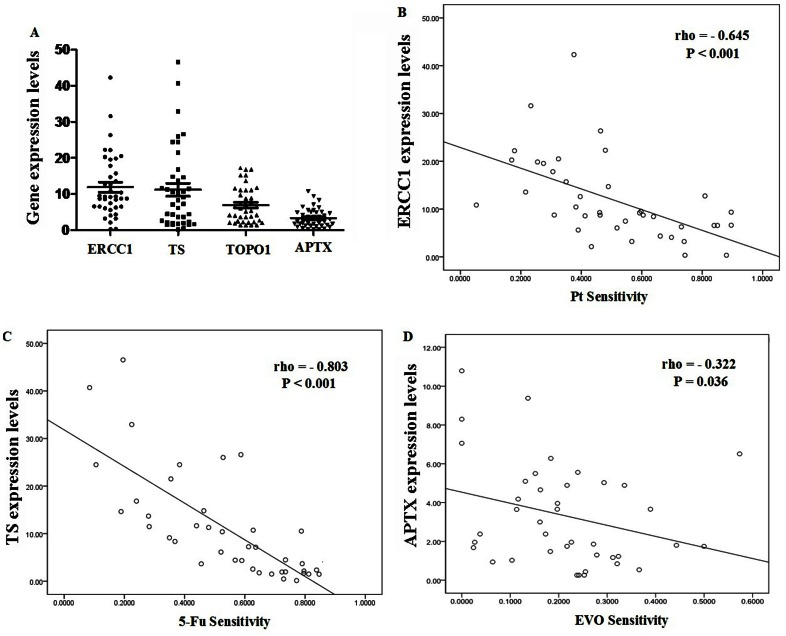
The mRNA expression levels of ERCC1,TS,TOPO1 and APTX, and their correlation with drug sensitivity. A, The mRNA expression levels of ERCC1,TS,TOPO1 and APTX. The lines in the middle stood for mean with SEM; n = 40. B, correlation between ERCC1 mRNA expression levels and Pt sensitivity (rho = −0.645, *P*<0.001, n = 40). C, correlation between TS mRNA expression levels and 5-FU sensitivity (rho = −0.803, *P*<0.001, n = 40). D, correlation between APTX mRNA expression levels and EVO sensitivity (rho = −0.322, *P = *0.036, n = 40).

To explore whether Pt, 5-FU and CPT-11 associated gene is involved in the anticancer effects of PPI and EVO on the freshly-removed gastric tumors, we first examined the mRNA expression levels of ERCC1, TS, TOPO1 and APTX in the samples before any drugs administration. The mRNA expression levels of the four genes were successfully detected in all the samples. The RT-PCR amplification curves were in Figure S1. The gene expression levels relative to housekeeping ACTB of 11.92 for ERCC1 (range 0.33–42.32, 95% confidence interval (CI): 9.12–14.72), 11.18 for TS (range 0.13–46.53, 95% CI: 7.56–14.80), 6.93 for TOPO1 (range 1.39–17.24, 95% CI: 5.34–8.52), and 3.33 for APTX (range 0.26–10.79, 95% CI: 2.49–4.17) (Figure 6A). There were strong significant correlation between ERCC1 mRNA expression levels and Pt sensitivity (*rho* = −0.645, *P*<0.001, Figure 6B), TS mRNA expression levels and 5-FU (*rho* = −0.803, *P*<0.001, Figure 6C). There were also weak but significant correlation between APTX mRNA expression levels and CPT-11 sensitivity (*rho* = −0.376, *P* = 0.017) or EVO sensitivity (*rho* = −0.322, *P* = 0.036, Figure 6D). However, the correlation between TOPO1 expression levels and CPT-11 sensitivity were not significant but only a trend (*rho* = 0.278, *P* = 0.082).

### The Effects of PPI and EVO on mRNA Expression Levels of Associated Genes

**Figure 7:**
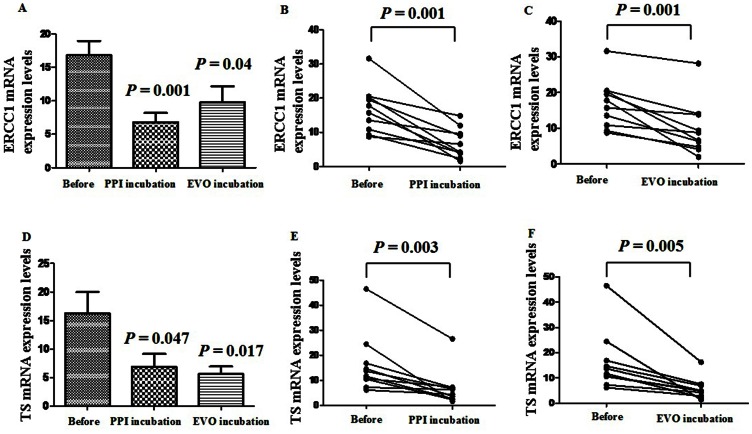
The effects of PPI and EVO on mRNA expression levels of ERCC1 and TS.

Next, we hypothesized that PPI and EVO might affect the expression of the investigated chemotherapeutic agents-associated genes in gastric cancers influencing sensitivity to those drugs. After incubation with PPI (200 µg/ml) or EVO (200 µg/ml) for 3days, mRNA expression levels of ERCC1, TS, TOPO1, and APTX in freshly-removed gastric tumors were assessed by quantitative RT-PCR. As shown in Figure 7, a significant change was observed in the mRNA expression levels of ERCC1 and TS. Most prominently, ERCC1 mRNA levels was markedly suppressed by the presentation of PPI (*P* = 0.001, n = 40) and slightly inhibited by the presentation of EVO (*P* = 0.04, n = 40, Figure 7A). Especially for ten samples with high mRNA expression levels of ERCC1, a significant decrease was observed after incubation with PPI or EVO (*P* = 0.001, respectively, Paired Student’s t test, Figure 7B–C). TS mRNA levels was also markedly suppressed by the presentation of EVO (*P* = 0.017) and slightly inhibited by the presentation of PPI (*P* = 0.047, Figure 7D). For ten samples with high mRNA expression levels of TS, a significant decrease was observed after incubation with PPI or EVO (*P* = 0.003, *P* = 0.005, respectively, Paired Student’s t test, Figure 7E–F). However, there were no significant changes of TOPO1 or APTX mRNA expression levels after incubation of PPI or EVO (data not shown). Although the mechanism of the interaction of PPI or EVO and chemotherapeutic drugs was not clear enough, it is notable that PPI and EVO could influence the expression of chemotherapeutic agent related genes, which might increase the sensitivity to these agents.

## Discussion

In the current study, we investigated the anticancer effects of two monomers extracted form Traditional Chinese Medicine (TCM) on freshly -removed tumor tissues. Our study demonstrated that PPI and EVO could enhance the anticancer effect of Pt and 5-FU, the baseline of chemotherapeutic agents for gastric cancer. The current study also showed that detection of ERCC1 and TS mRNA expression levels would be helpful to predict the possible benefit from the Pt and 5-FU based chemotherapy regime; meanwhile, APTX might be a candidate predictive biomarker for EVO. Moreover, the mechanism of PPI and EVO enhancement of the anticancer effect of Pt and 5-FU in gastric tumor might be due to their ability of reduce the mRNA expression levels of ERCC1 and TS.

PPT and EVO are two small molecular monomer extracted from TCM, which have been reported to inhibit cell activity and induce cell death in several types of cancer cell lines. However, to our best knowledge, rare study has reported their anticancer effect on freshly-removed tumor tissues. The current study showed that both PPI and EVO could inhibit the activity of fresh tumor tissues with the median inhibition rates of 20.64%±14.25% for PPI and 21.14%±13.43% for EVO. We have to admit that these inhibition rates are not high enough for PPI and EVO to be used alone in clinical practice. Thus, we further study their effects on promotion the anticancer efficiency of three most commonly used chemotherapeutic agents in gastric cancer therapy. Fortunately, the results showed that PPI could not only increase the Pt and 5-FU sensitivity of the freshly-removed tumor, but also could reverse the Pt resistance; EVO could significantly increase 5-FU sensitivity of the freshly-removed tumor.

ERCC1 plays a key role in the DNA repair pathway, and has been demonstrated that lung and gastric cancer patients with lower mRNA expression levels of ERCC1 could benefit from platinum-based chemotherapy[21]–[23]. Cell lines and clinical study also reported that mRNA expression levels of TS are associated with 5-FU sensitivity [24]–[27]. In agree with the previous study, our data also showed that there were significant correlations between ERCC1 mRNA expression levels and Pt sensitivity, and between TS mRNA expression levels and 5-FU sensitivity. Based on this, we hypothesized that PPI and EVO might affect the mRNA expression of these genes in gastric cancers influencing sensitivity to those drugs. By comparing the ERCC1 and TS mRNA expression levels before and after PPI or EVO incubation, it showed that ERCC1 and TS mRNA expression levels both reduced significantly after the effect of PPI and EVO, which partially explained the reason why those two molecular monomers could increase the sensitivity to these agents.

Topoisomerase1 (TOPO1) regulates DNA supercoiling during replication by causing single-strand breaks and relegation^.^ It has been reported that EVO could inhibit type TOPO1 and II exhibiting enhanced inhibition against camptothecin (CPT) resistant human leukaemia cells [8]. However, in the current study, there was no significant enhanced inhibition on freshly-removed gastric tumor tissues when combined EVO and CPT-11 compared with CPT-11 alone. There was no significant correlation between TOPO1 mRNA expression levels and CPT-11 or EVO sensitivity, either. The differences in cancer types and the heterogeneity of freshly-removed tumor tissues may account for the differentiation between our results and previous study. For further validation, gastric cell line study needs to be carried out and more freshly-removed samples need to be included and analyzed.

Aprataxin (APTX) is a protein in the histidine triad domain super family involved in the repair of single-stranded DNA strand breaks. It has been reported that colon cancer patients with lower levels of APTX might be more sensitive to CPT-11 based chemotherapy [28]. In the current study, a weak but significant correlation was observed between APTX mRNA expression levels and CPT-11 sensitivity or EVO sensitivity, which means APTX may be a possible predictive biomarker for EVO sensitivity. Therefore, we hypotheses that patient with low mRNA expression level of APTX may be more sensitive to EVO. This finding is novel and has not been reported ever before. Nevertheless, the hypothesis needs to be explored by deeper studies such as the mRNA and protein expression of APTX in the EVO-sensitive and EVO-resistant cells. We will also use siRNA to confirm the relationship between EVO sensitivity and APTX mRNA expression level.

The chemosensitivity assay we adopted here included HDRA for *in vitro* testing. HDRA has been demonstrated by varieties of studies as a useful predictor for chemosensitivity at different cancerous sites, including gastrointestinal cancer [29]. The collagen sponge-gel-supported histoculture conserves the original phenotypic characteristics and potential cellular interactions of tumor cells [16], and highly mimics the growth of the tumors *in vivo* [30]. It has been reported in gastric cancer [29], esophageal cancer [16], breast cancer [31], oral squamous cell carcinomas [32] and head and neck cancer [33] that efficacy rate for an individual agent using HDRA assay *in vitro* has a considerable good correlation with clinical response rate to each agent. We designed 8 parallel culture wells for sensitivity testing and 8 parallel culture wells for control from different parts of one patient’s tumor sample avoiding tumor heterogeneity. We have recognized that any kind of *in vitro* may have defects, thus, more adequately designed clinical trials will be carried out for further confirmation.

In conclusion, this study provided evidence for PPI and EVO to be useful assistants for Pt and 5-FU based chemotherapy. Patients with gastric cancer receiving this regimen may get benefit from the administration of PPI and EVO included TCM therapy.

## Supporting Information

Figure S1 **The RT-PCR amplification curves of different genes.**
(TIF)

Table S1 **The effects of PPI and EVO on reverse Pt resistance at different doses.**
(DOC)
