# Supplementary figures and images for: Synergistic Anticancer Effects of Polyphyllin I and Evodiamine on Freshly-Removed Human Gastric Tumors
Source: PLoS One. 2013 Jun 7;8(6):e65164. doi: 10.1371/journal.pone.0065164 (PMC3676398; doi:10.1371/journal.pone.0065164)

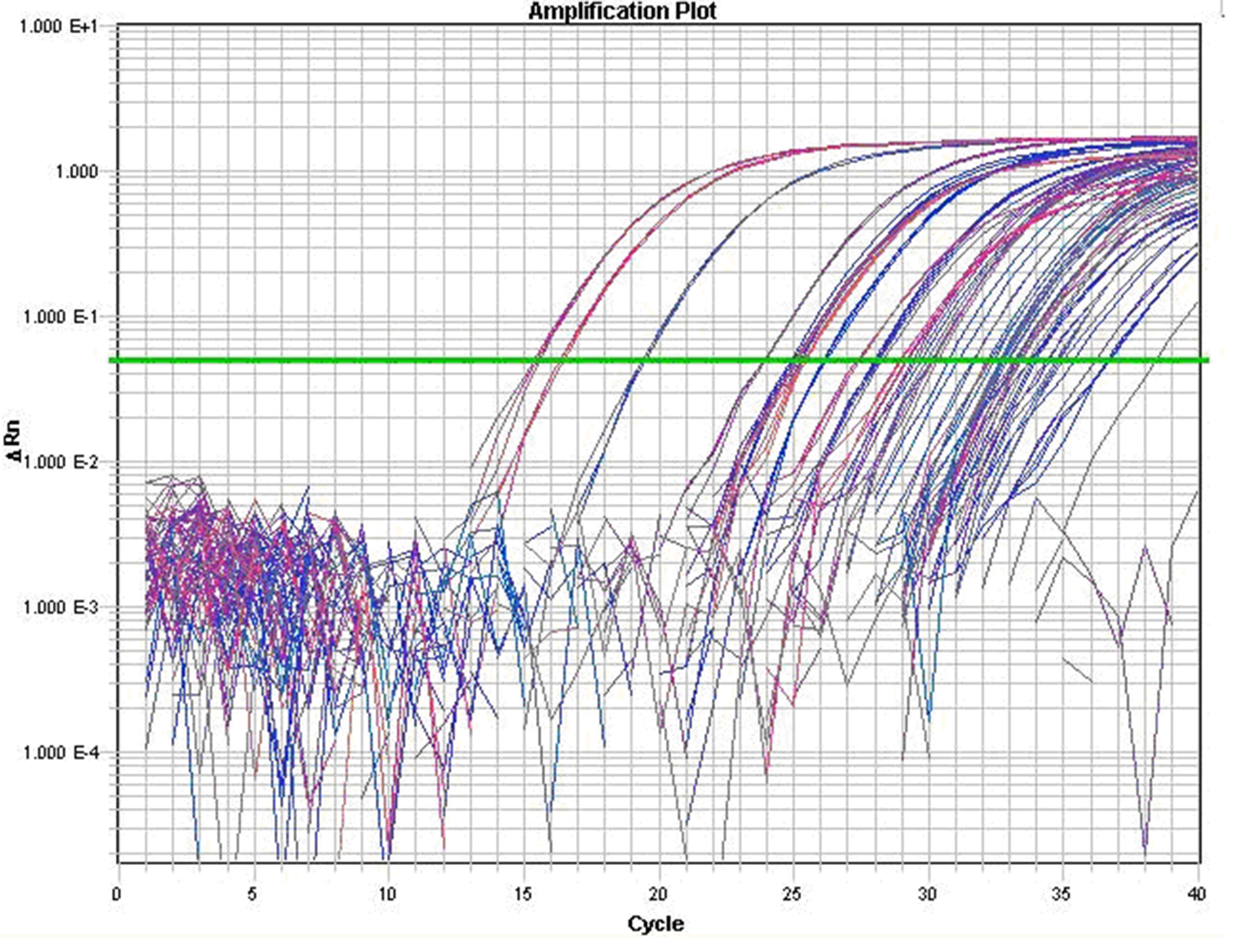

Supplement: Figure S1 — The RT-PCR amplification curves of different genes. (TIF) [file pone.0065164.s001.tif]
