# Supplementary material for: Synergistic Anticancer Effects of Polyphyllin I and Evodiamine on Freshly-Removed Human Gastric Tumors
Source: PLoS One. 2013 Jun 7;8(6):e65164. doi: 10.1371/journal.pone.0065164 (PMC3676398; doi:10.1371/journal.pone.0065164)
Supplement: Table S1 — The effects of PPI and EVO on reverse Pt resistance at different doses. (DOC) [file pone.0065164.s002.doc]

**Table S1 The effects of PPI and EVO on reverse Pt resistance at different doses**

| Patients | Pt (20μg/ml) | Pt+PPI  (50μg/ml) | Pt+PPI  (100μg/ml) | Pt+PPI  (200μg/ml) | Pt+EVO  (50μg/ml) | Pt+EVO  (100μg/ml) | Pt+EVO  (200μg/ml) |
| --- | --- | --- | --- | --- | --- | --- | --- |
| P1 | 27.49% | 33.18% | 40.88% | 47.61% | 29.10% | 30.80% | 34.97% |
| P4 | 30.60% | 38.77% | 40.12% | 46.70% | 30.66% | 30.70% | 30.99% |
| P8 | 32.45% | 39.42% | 50.22% | 50.86% | 42.76% | 60.00% | 61.62% |
| P9 | 34.99% | 40.36% | 48.73% | 61.12% | 40.50% | 42.40% | 43.94% |
| P11 | 21.58% | 50.13% | 68.54% | 70.03% | 21.90% | 22.10% | 22.76% |
| P12 | 5.28% | 20.28% | 20.89% | 24.53% | 30.22% | 36.12% | 38.73% |
| P13 | 23.31% | 38.02% | 38.90% | 39.17% | 29.20% | 31.87% | 32.89% |
| P15 | 25.51% | 28.74% | 30.03% | 31.41% | 28.43% | 25.77% | 19.48% |
| P18 | 16.98% | 20.42% | 24.39% | 26.34% | 29.35% | 32.56% | 36.80% |
| P20 | 31.05% | 44.19% | 49.10% | 52.89% | 35.73% | 38.79% | 41.63% |
